# Longevity in Bovids Is Promoted by Sociality, But Reduced by Sexual Selection

**DOI:** 10.1371/journal.pone.0045769

**Published:** 2012-09-21

**Authors:** Jakob Bro-Jørgensen

**Affiliations:** Mammalian Behaviour & Evolution Group, Department of Evolution, Ecology & Behaviour, Institute of Integrative Biology, Faculty of Health and Life Sciences, University of Liverpool, Neston, United Kingdom; University of Manitoba, Canada

## Abstract

Selection on intrinsic lifespan depends on both external factors affecting mortality and inherent tradeoffs in resource allocation between viability traits and other fitness-related traits. Longevity is therefore likely to vary between species in a sex-specific manner due to interspecific and intersexual differences in behavioural ecology. Here I focus on the bovid family to test two central hypotheses on longevity selection using the comparative method: firstly, that a reduction of extrinsic mortality in social species strengthens selection on intrinsic lifespan, and secondly, that mortality costs associated with intense sexual selection lead to shorter intrinsic lifespan. The results show that longevity (*i*) increases with sociality in both sexes and (*ii*) decreases with male-biased sexual size-dimorphism, but in males only. These discoveries suggest that sociality, a key ungulate strategy to reduce predation-related mortality, selects for inherently longer-lived organisms, and that strong sexual selection, which is known to compromise survival rates in the wild, can constrain also intrinsic lifespan. The contrasting results for males and females indicate that selection on longevity in the two sexes is partly uncoupled.

## Introduction

The extent to which intrinsic lifespan is visible to selection depends on extrinsic mortality rates due to environmental conditions [1]. Where mortality from external factors, such as predation, is high, intrinsic lifespan is less likely to be expressed and thus subjected to selection; by contrast, low extrinsic mortality intensifies selection on longevity [2]–[4]. A key strategy to reduce predation in many taxa is the formation of groups, which may both enhance vigilance against predators (‘detection effect’ [5]) and reduce the probability of being preyed upon, should a predator attack (‘dilution effect’ [6]). Yet, where group-living has evolved as an antipedator strategy, the association between sociality and predation rate at the interspecific level is not straightforward, and empirical investigation is therefore essential to establish how longevity relates to sociality. Two scenarios as to how sociality and longevity are linked can be envisaged. On one hand, selection for longevity may be strongest in social species if the antipredator benefits of group-living result in lower extrinsic mortality compared to less social species. On the other hand, sociality may have evolved primarily where predation rates are high, and if the antipredator benefits of group-living only partly offset the higher extrinsic mortality, positive selection on longevity may be less intense in social species.

Intrinsic lifespan may also depend crucially on the intensity of sexual selection [7]. In natural environments, sexually selected traits can increase vulnerability to extrinsic mortality factors directly, e.g. where increased conspicuousness or reduced manoeuvrability enhances predation risk, and this may attenuate selection on intrinsic lifespan as described above. Also, investment in sexually selected traits may reduce longevity due to inherent tradeoffs in resource allocation against investment in longevity-promoting traits. Studies have found that in species where males are under stronger sexual selection than females, they exhibit higher rates of senescence in certain traits in the wild [8], [9] and also invest less in morphological traits linked to viability [10], [11]. These findings highlight the possibility of a general link between sexual selection and shorter intrinsic lifespan.

I here adopt the comparative method with phylogenetic control to investigate the impact of sociality and sexual selection on the evolution of intrinsic lifespan, using the family Bovidae as a model taxon. Bovids were chosen as the study system not only because they provide a classical example of a taxon where sociality has evolved due to anti-predator benefits [12], [13], but also because this taxon is speciose and demonstrates pronounced interspecific variation in both sociality and the intensity of sexual selection.

**Figure 1:**
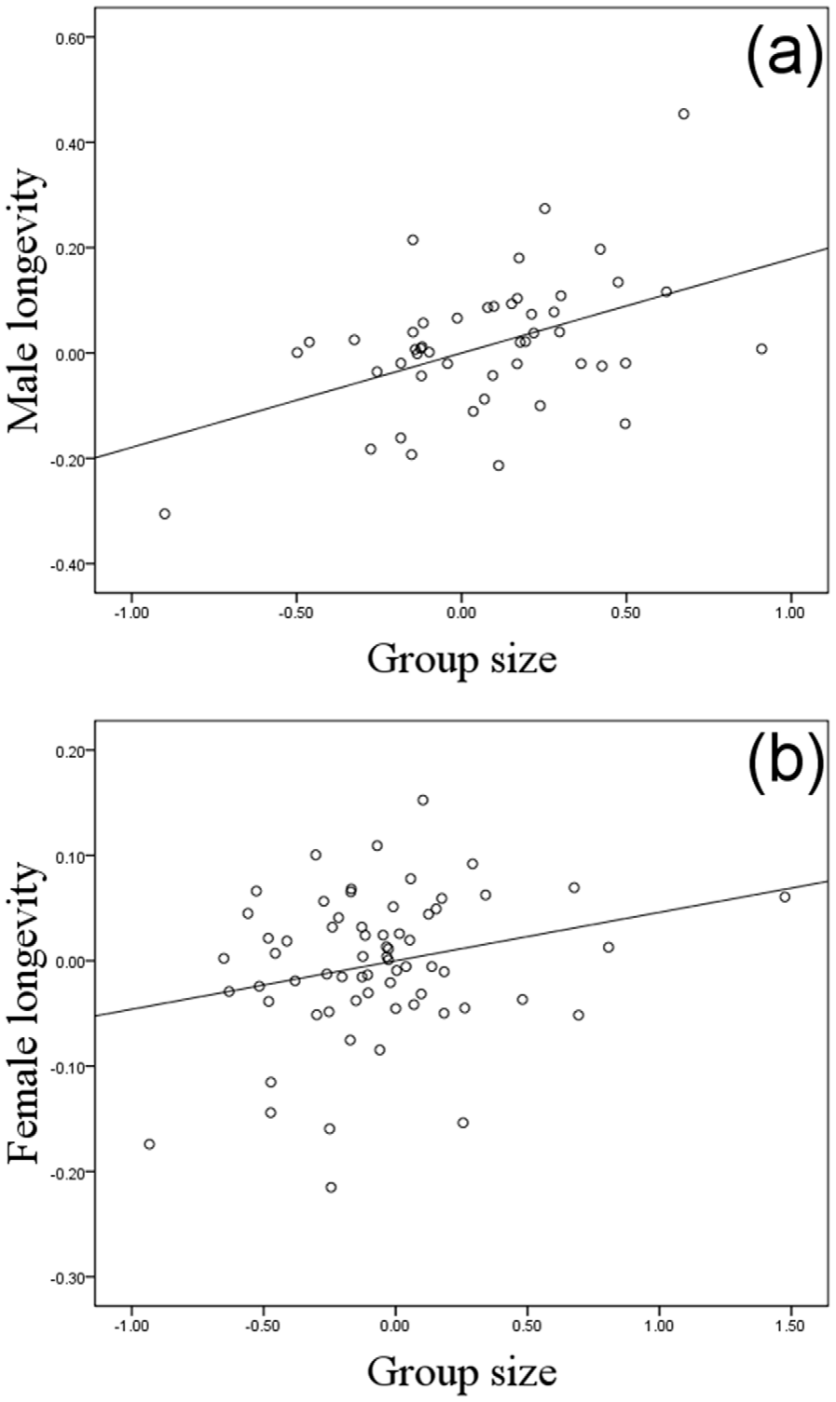
Partial regression plots of contrasts in longevity against contrasts in group size. (a) Males. (b) Females. The lines show the significant slopes from linear regression through the origin.

**Figure 2:**
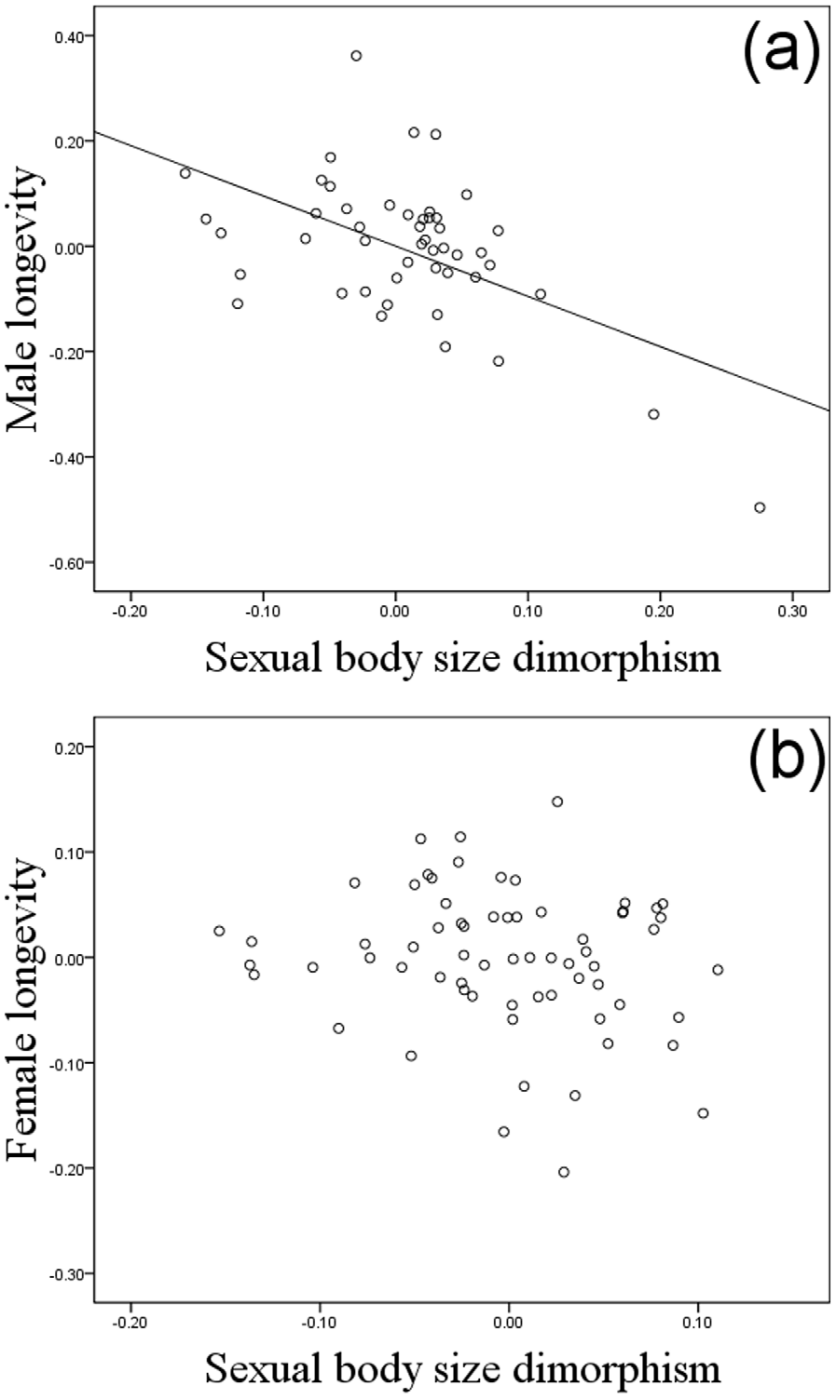
Partial regression plots of contrasts in longevity against contrasts in sexual body size dimorphism. (a) Males. (b) Females. The line shows the significant slope from linear regression through the origin.

**Figure 3:**
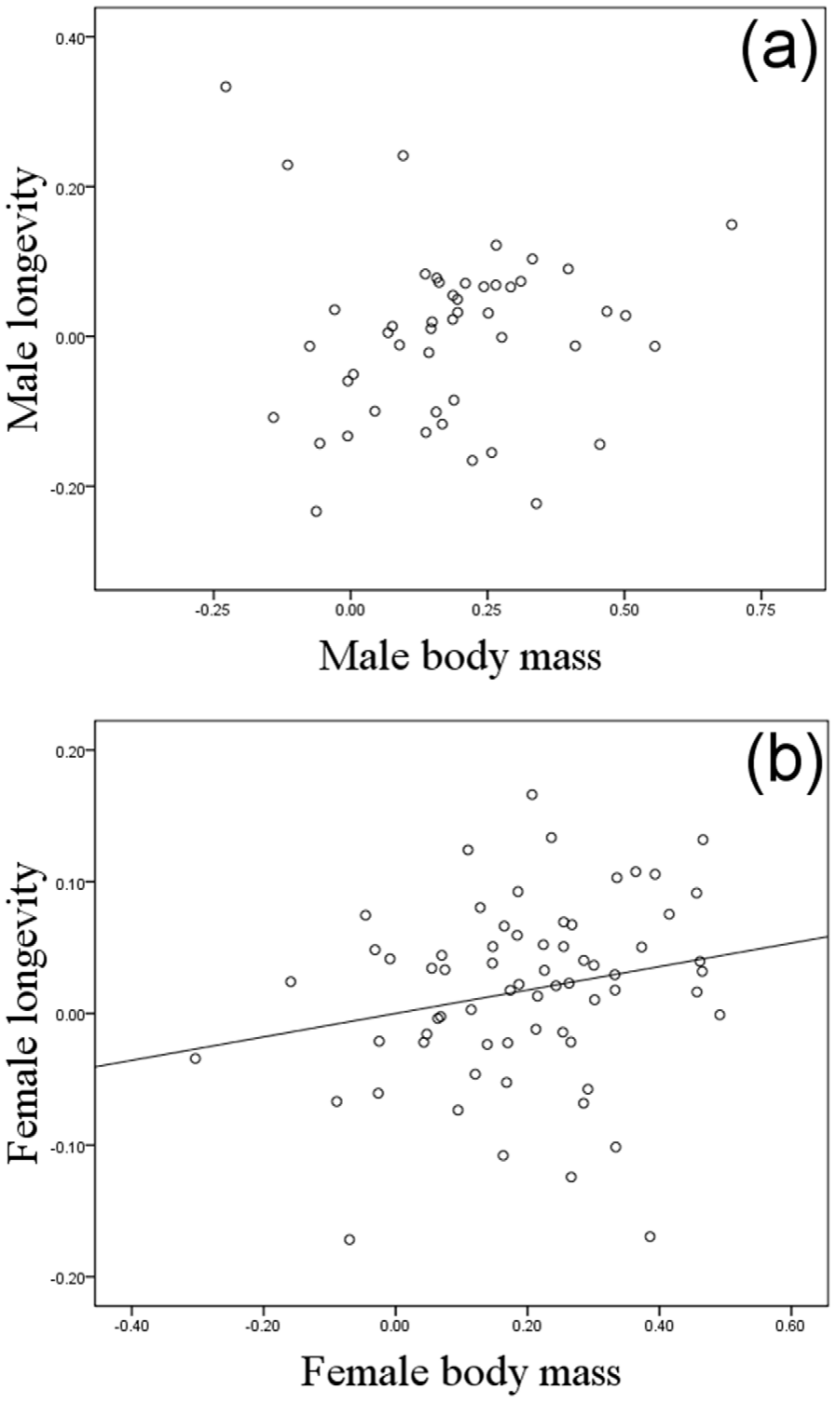
Partial regression plots of contrasts in longevity against contrasts in sex-specific body mass. (a) Males. (b) Females. The line shows the significant slope from linear regression through the origin.

**Figure 4:**
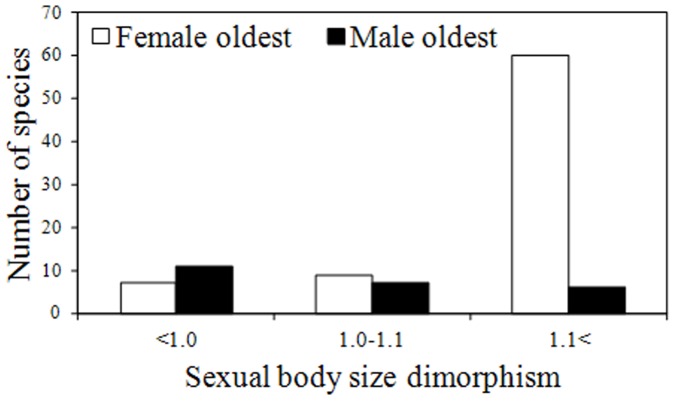
The number of species in which a male respectively a female holds the longevity record; shown in relation to the degree of sexual body size dimorphism.

**Table 1:** Model of longevity in male bovids (59 species, 48 contrasts; *r* ^2^ = 0.344, F = 12.04, df 2,46, P<0.001).

	B (±SE)	β	df	*t*	P	VIF
Sexual body size dimorphism	−0.954±0.219	−0.550	2,46	−4.356	<0.001	1.120
Group size	0.179±0.050	0.449	2,46	3.553	0.001	1.438
Male body mass	0.018±0.066	0.038	3,45	0.269	0.789	1.338
Interaction group size:malebody mass	0.0351±0.0318	–	4,44	1.105	0.275	–

**Table 2:** Model of longevity in female bovids (87 species, 66 contrasts; *r* ^2^ = 0.245, F = 10.4, df 2,64, P<0.001).

	B (±SE)	β	df	*t*	P	VIF
Female body mass	0.089±0.032	0.331	2,64	2.75	0.008	1.226
Group size	0.046±0.022	0.253	2,64	2.105	0.039	1.260
Sexual body size dimorphism	−0.196±0.136	−0.158	3,63	−1.443	0.154	1.037
Interaction group size:femalebody mass	−0.0068±0.0174	–	4,62	−0.390	0.698	–

First, I test the two hypotheses on the association between sociality and longevity. If antipredator benefits of group-living lead to positive selection on intrinsic lifespan in social species due to reduced extrinsic mortality, I predict that longevity will increase with group size. However, if sociality evolves primarily under intense predation and remains associated with high extrinsic mortality in spite of antipredator benefits from group-living, I predict that longevity will decrease with group size. Next, I investigate the hypothesis that sexual selection has a negative impact on intrinsic lifespan. As a proxy measure of the intensity of sexual selection, I use sexual body size dimorphism (SSD), a key sexually selected trait in bovids, whose evolution is linked to the advantage that large body size confers in contests over mates [14]–[16]. Thus, if viability costs of sexual selection constrain the evolution of intrinsic lifespan, I predict that longevity will decrease with increasing sexual size dimorphism. To disentangle the sex-specific evolutionary pressures, I conduct the analyses on the two sexes separately. In the analyses, I control for the effect of body size on longevity [17]. In particular, when investigating the link between sociality and longevity, I also take into account that the benefit of sociality as an antipredator strategy is thought to depend on body size: whereas larger species typically live in open habitats and rely on safety in numbers, minimal group size is believed to be advantageous in smaller species, which usually live in closed habitats and hide from predators [12], [13].

## Materials and Methods

### Data

Focusing on all extant bovid species, data was collected from the literature on the maximum recorded lifespans of males and females. These records invariably came from captive populations and were assumed to broadly reflect intrinsic longevity, i.e. independent of ecologically induced mortality due to predation, food limitation, mate competition and disease transmission [18]. Data was also compiled on average body masses of adult males and females, as well as on average breeding group size. Since data was lacking for some species, the final data set was restricted to 100 out of a total of 136 species. SSD was calculated as the Lovich & Gibbons ratio (i.e. M/F if M>F and 2-F/M if F>M, where M and F denote male and female body mass, respectively [19]). All variables were log_10_-transformed to make them suitable for regression analysis. The data on maximum longevity was obtained primarily from [20], and supplemented from [21]–[24]. The data on the morphological and behavioural variables was copied from [16], [25], and supplemented from [13], [26]–[28]. The data set is presented in Table S1.

### Comparative Analysis

The comparative method with phylogenetic control was used to identify predictors of longevity [29]. Evolutionarily independent contrasts were calculated in R [30] using the packages caper [31] and ape [32]. Phylogenetic relatedness was controlled for by assuming a gradual model of evolution based on the phylogenetic supertree and branch lengths in [33]. The independent contrasts were computed using the crunch-algorithm which assumes that trait evolution can be modelled as a random walk process [34]. To test this assumption, the estimated contrasts were regressed against their estimated nodal values [31]. I also tested the success of the contrast standardization procedure by regressing the absolute values of the standardized contrasts against the estimated standard deviation at each node, thereby assessing the heterogeneity of variance in the residuals and the suitability of the data for regression analysis [31]. As expected given the assumptions, no significant associations were found in any of these tests, which were all performed in R.

Contrasts in both male and female longevity followed a normal distribution, and I performed separate multivariate regression analysis on the two sexes in PASW Statistics 17.0.2. I tested the predictive power of the following independent variables: sex-specific body mass, group size, and SSD. Because antipredator strategy in ungulates depends on body size (see Introduction), the interaction between body mass and group size was also included as an independent variable in order to assess whether any effect of sociality on longevity was size-dependent. All regressions were through the origin. Non-significant variables were removed by backward elimination. Due to correlations between the independent variables, I assessed the potential effect of collinearity on the results of the multivariate analyses by calculating the variance inflation factors. In all cases, the variance inflation factors were <1.5 and thus well below the commonly accepted critical thresholds for significant collinearity of 5–10 [35]. Performing the analysis using the phylogenetic generalized least squares (PGLS) method yielded similar results [36]. Also, restricting the analyses to the subset of 46 species for which longevity records on both sexes were available revealed the same patterns, except that the positive association between body mass and longevity in females was not significant.

## Results

Longevity in both males and females was positively correlated with group size across the bovid species (Tables 1 and 2, Figure 1); however, no effect was found of the interaction between group size and body size in either sex (Tables 1 and 2). Sexual size dimorphism showed an independent, negative, correlation with longevity in males but not in females (Tables 1 and 2, Figure 2). In females, longevity was positively related to body size, a pattern not found in males (Tables 1 and 2, Figure 3). In 24% of species, a male rather than a female held the longevity record, and these species were significantly less size-dimorphic (SSD where the record holder was male: 1.10±0.06, as compared to female: 1.39±0.04, mean±SE; *t* = 4.26, df 98, P<0.001; Figure 4).

## Discussion

This study reveals both sociality and sexual selection (as measured by SSD) as independent predictors of interspecific variation in longevity in bovids. Although the intensity of sexual selection is partly dependent on sociality [16], the multivariate approach taken here allows the distinct, oppositely directed effects of these two factors to be teased apart by analysing sex-specific relationships.

The negative relationship between male longevity and SSD suggests that in species where the fitness of males is strongly linked to their success in mate competition, viability costs associated with the development of sexually selected traits constrain selection on male lifespan. This interpretation is consistent with studies of wild populations, which show male survival rates to be compromised by intense mate competition [37]–[40]: such higher mortality is indeed expected to attenuate selection for long intrinsic lifespan. Further evidence that relatively short lifespan is not a general male characteristic but depends specifically on the intensity of sexual selection on males, is the finding that it is males rather than females, who tend to hold the longevity record in species where size dimorphism is female-biased. That the longevity of males not only equals, but generally supersedes that of females in this case, suggests that selection on lifespan in these species is more constrained by the costs of maternal investment than by the costs of reproductive competition. Again, this is consistent with survival rates in the wild as mortality in monogamous mammals is reported to be biased towards females in general [37].

The positive link between longevity and group size supports the hypothesis that selection for long intrinsic lifespan is stronger in social species due to lower extrinsic mortality from predation as a result of safety in numbers [1], [3]. Apart from the classical detection and dilution benefits of group living, it is conceivable that the positive link between sociality and longevity is promoted also by a general link between large brain size and sociality [1], which has been reported specifically in ungulates [41], [42]. The primary driver of large brain size evolution in social species is thought to be the need to cope with more complex inter-individual relations [41], but larger brains may also result in reduced mortality by enabling organisms to respond more appropriately to dangerous situations. A role of increased brain size in mediating the positive link between sociality and longevity could explain the somewhat surprising finding that the longevity-promoting effect of sociality was independent of body size: if group-living selects for increased longevity solely owing to detection and dilution effects, the link would be expected mainly in larger species since smaller ungulates typically rely on concealment in groups of minimal size to avoid predation. However, further investigation is required to disentangle the exact causal relationships between sociality, predation rates and longevity.

The finding that social life is associated with increased longevity in bovids contrasts with a recent study, which as part of a broader investigation examined non sex-specific longevity in a subset of Artiodactyl species (71 of 233 spp., i.e. 30%) [43]. Contrary to their expectation, the authors found a negative rather than a positive association. I propose that the discrepancy between the two studies can be explained by (*i*) the use of a more representative and comprehensive data set in the present study (100 of 136 bovid spp., i.e. 74%), and (*ii*) correction of errors in group sizes in the data set used in the former analysis (e.g. the present study uses a group size of 2 rather than 6.25 for the monogamous Guenther’s dikdik *Madoqua guentheri*, and 6 rather than 1 for the social dama gazelle *Gazella dama*; see Table S1 for further details).

In summary, the positive link between longevity and sociality, a key antipredator strategy in ungulates, points to lower extrinsic mortality in social species as a factor selecting for inherently longer lifespan. At the same time, the negative link between male longevity and SSD, a key sexually selected trait, suggests that investment in reproductive fitness components may not only compromise survival in the wild but also constrain selection on intrinsic longevity. Now further studies are needed to establish the critical conditions under which these selective forces have had a significant impact on longevity selection.

## Supporting Information

Table S1 The data set.
(DOCX)
