# Supplementary material for: Longevity in Bovids Is Promoted by Sociality, But Reduced by Sexual Selection
Source: PLoS One. 2012 Sep 21;7(9):e45769. doi: 10.1371/journal.pone.0045769 (PMC3448691; doi:10.1371/journal.pone.0045769)
Supplement: Table S1 — The data set. (DOCX) [file pone.0045769.s001.docx]

**Table S1.** The data set (for references, see ‘Materials and methods’).

| Species | Male body mass (kg) | Female body mass (kg) | Sexual body size dimorphism | Group size | Male longevity (yrs) | Female longevity (yrs) |
| --- | --- | --- | --- | --- | --- | --- |
| *Addax nasomaculatus* | 117.7 | 84.7 | 1.39 | 20 | - | 28.0 |
| *Aepyceros melampus* | 56.9 | 43.8 | 1.30 | 20 | - | 25.6 |
| *Alcelaphus buselaphus* | 178.3 | 162.7 | 1.10 | 10 | 17.6 | 22.5 |
| *Ammotragus lervia* | 111.8 | 51.6 | 2.17 | 3 | - | 21.7 |
| *Antidorcas marsupialis* | 40.7 | 35.5 | 1.15 | 24 | - | 19.8 |
| *Antilope cervicapra* | 40.2 | 34.4 | 1.17 | 23 | 22.7 | 23.9 |
| *Beatragus hunteri* | 91.0 | 86.0 | 1.06 | 18 | 15.2 | - |
| *Bison bison* | 795.3 | 452.8 | 1.76 | 57 | 33.5 | 29.5 |
| *Bison bonasus* | 718.0 | 423.0 | 1.70 | 20 | 17.5 | 26.4 |
| *Bos gaurus* | 848.4 | 701.9 | 1.21 | 9.5 | 14.9 | 26.2 |
| *Bos javanicus* | 750.0 | 450.0 | 1.67 | 21 | 18.3 | 27.1 |
| *Bos mutus* | 590.5 | 306.0 | 1.93 | 80 | - | 26.3 |
| *Boselaphus tragocamelus* | 253.3 | 136.3 | 1.86 | 7 | - | 21.7 |
| *Bubalus arnee* | 1200.0 | 800.0 | 1.50 | 21 | 29.2 | 34.9 |
| *Bubalus depressicornis* | 156.0 | 145.0 | 1.08 | 1 | 36.1 | 33.0 |
| *Budorcas taxicolor* | 282.7 | 160.0 | 1.77 | 22.5 | 20.5 | 21.9 |
| *Capra aegagrus* | 53.0 | 38.6 | 1.37 | 15 | - | 20.8 |
| *Capra caucasica* | 86.3 | 55.0 | 1.57 | 12 | - | 19.3 |
| *Capra cylindricornis* | 82.8 | 50.0 | 1.66 | 12 | - | 20.2 |
| *Capra falconeri* | 92.7 | 36.3 | 2.55 | 9 | 10.5 | 19.1 |
| *Capra ibex* | 80.5 | 48.9 | 1.65 | 15 | - | 20.9 |
| *Capra nubiana* | 70.0 | 60.0 | 1.17 | 20 | - | 22.4 |
| *Capra pyrenaica* | 72.5 | 40.0 | 1.81 | 10 | 14.2 | 15.2 |
| *Capra sibirica* | 90.0 | 44.2 | 2.04 | 13.8 | - | 22.3 |
| *Capricornis crispus* | 35.9 | 38.4 | 0.93 | 1 | 20.9 | 24.0 |
| *Capricornis sumatraensis* | 121.0 | 119.0 | 1.02 | 1 | 18.5 | 14.6 |
| *Cephalophus dorsalis* | 20.3 | 19.5 | 1.04 | 1 | 17.1 | 17.5 |
| *Cephalophus jentinki* | 70.3 | 80.5 | 0.87 | 1 | 21.0 | - |
| *Cephalophus natalensis* | 13.1 | 12.1 | 1.08 | 2 | 15.2 | - |
| *Cephalophus niger* | 19.9 | 18.4 | 1.08 | 1 | 14.8 | - |
| *Cephalophus nigrifrons* | 13.3 | 15.0 | 0.89 | 1 | 17.9 | - |
| *Cephalophus rufilatus* | 10.1 | 10.3 | 0.98 | 1 | 14.3 | 15.2 |
| *Cephalophus silvicultor* | 52.5 | 72.0 | 0.63 | 2 | 22.5 | 21.5 |
| *Cephalophus zebra* | 13.9 | 14.7 | 0.94 | 1 | 13.0 | 11.8 |
| *Connochaetes gnou* | 166.7 | 135.0 | 1.23 | 21 | - | 21.8 |
| *Connochaetes taurinus* | 235.3 | 184.9 | 1.27 | 15 | 21.4 | 24.3 |
| *Damaliscus lunatus* | 137.0 | 120.1 | 1.14 | 6 | 15.8 | 23.6 |
| *Damaliscus pygargus* | 62.8 | 61.2 | 1.03 | 8 | 16.5 | 23.0 |
| *Eudorcas rufifrons* | 27.0 | 20.7 | 1.30 | 5 | 10.8 | 14.5 |
| *Eudorcas thomsonii* | 22.7 | 17.8 | 1.28 | 28 | 19.9 | 20.0 |
| *Gazella cuvieri* | 29.4 | 20.4 | 1.44 | 4.5 | 17.9 | - |
| *Gazella dorcas* | 16.3 | 13.3 | 1.23 | 17 | 18.7 | 23.7 |
| *Gazella gazella* | 23.3 | 20.8 | 1.12 | 6 | - | 15.3 |
| *Gazella leptoceros* | 27.2 | 20.9 | 1.30 | 6 | - | 14.6 |
| *Gazella spekei* | 21.3 | 16.6 | 1.28 | 8 | - | 14.0 |
| *Gazella subgutturosa* | 27.4 | 23.2 | 1.18 | 4 | - | 16.3 |
| *Hemitragus jemlahicus* | 103.3 | 56.0 | 1.84 | 15 | - | 21.8 |
| *Hippotragus equinus* | 274.4 | 256.4 | 1.07 | 13 | 17.2 | 25.9 |
| *Hippotragus niger* | 235.2 | 216.6 | 1.09 | 20 | - | 22.2 |
| *Kobus ellipsiprymnus* | 236.8 | 187.3 | 1.26 | 8 | 21.0 | 30.0 |
| *Kobus kob* | 97.5 | 61.9 | 1.58 | 25 | - | 21.9 |
| *Kobus leche* | 104.3 | 78.7 | 1.33 | 12 | - | 25.4 |
| *Kobus megaceros* | 105.5 | 75.0 | 1.41 | 20 | - | 21.1 |
| *Litocranius walleri* | 35.0 | 34.3 | 1.02 | 3 | 16.5 | 17.3 |
| *Madoqua guentheri* | 3.7 | 4.5 | 0.78 | 2 | - | 17.5 |
| *Madoqua kirkii* | 4.6 | 5.1 | 0.89 | 2 | 17.3 | 18.5 |
| *Naemorhedus caudatus* | 31.7 | 32.3 | 0.98 | 8 | 20.3 | 19.3 |
| *Naemorhedus goral* | 32.0 | 29.9 | 1.07 | 8 | 17.6 | - |
| *Nanger dama* | 68.0 | 51.0 | 1.33 | 6 | - | 19.3 |
| *Nanger granti* | 72.1 | 46.0 | 1.57 | 10 | - | 19.7 |
| *Nanger soemmerringii* | 45.4 | 33.6 | 1.35 | 7 | 14.1 | 15.5 |
| *Neotragus moschatus* | 4.8 | 5.1 | 0.94 | 1.5 | 13.5 | 13.1 |
| *Neotragus pygmaeus* | 2.0 | 2.2 | 0.91 | 1.5 | 11.1 | - |
| *Nilgiritragus hylocrius* | 100.0 | 50.0 | 2.00 | 7 | - | 17.3 |
| *Oreamnos americanus* | 95.9 | 61.0 | 1.57 | 4 | 14.0 | 20.8 |
| *Oreotragus oreotragus* | 11.3 | 13.0 | 0.85 | 2 | 20.0 | 25.9 |
| *Oryx beisa* | 178.0 | 166.4 | 1.07 | 23 | 21.1 | 23.8 |
| *Oryx dammah* | 165.0 | 150.0 | 1.10 | 12 | - | 27.5 |
| *Oryx leucoryx* | 85.9 | 80.1 | 1.07 | 15 | - | 20.8 |
| *Ourebia ourebi* | 14.1 | 15.1 | 0.93 | 3 | 15.9 | 10.5 |
| *Ovibos moschatus* | 356.0 | 247.3 | 1.44 | 15 | - | 27.4 |
| *Ovis ammon* | 120.7 | 63.2 | 1.91 | 50 | - | 16.8 |
| *Ovis canadensis* | 83.4 | 58.7 | 1.42 | 10 | 16.7 | 20.6 |
| *Ovis dalli* | 80.4 | 53.3 | 1.51 | 3.7 | - | 19.6 |
| *Ovis nivicola* | 103.2 | 52.9 | 1.95 | 17.5 | 10.8 | - |
| *Ovis orientalis* | 55.0 | 36.4 | 1.51 | 63 | - | 22.8 |
| *Pelea capreolus* | 24.0 | 25.0 | 0.96 | 4 | - | 12.3 |
| *Philantomba maxwellii* | 6.5 | 7.2 | 0.89 | 1.5 | 18.3 | - |
| *Philantomba monticola* | 4.4 | 5.1 | 0.84 | 2 | 15.9 | 15.3 |
| *Procapra gutturosa* | 31.5 | 24.0 | 1.31 | 20 | 12.0 | 9.6 |
| *Pseudois nayaur* | 60.0 | 39.5 | 1.52 | 11.5 | - | 20.9 |
| *Raphicerus campestris* | 10.9 | 11.3 | 0.96 | 1.5 | 9.3 | - |
| *Raphicerus melanotis* | 10.7 | 10.5 | 1.02 | 1 | - | 7.7 |
| *Redunca arundinum* | 58.3 | 43.2 | 1.35 | 3 | 16.8 | - |
| *Redunca fulvorufula* | 30.1 | 28.5 | 1.06 | 4 | 15.0 | 14.0 |
| *Redunca redunca* | 51.6 | 40.3 | 1.28 | 4 | 10.3 | 10.7 |
| *Rupicapra rupicapra* | 40.3 | 31.7 | 1.27 | 20 | - | 17.6 |
| *Saiga tatarica* | 42.5 | 32.3 | 1.32 | 35 | 10.5 | 10.0 |
| *Sylvicapra grimmia* | 18.3 | 19.6 | 0.93 | 2 | 14.3 | 15.4 |
| *Syncerus caffer* | 642.9 | 467.5 | 1.38 | 50 | 28.2 | 29.8 |
| *Tetracerus quadricornis* | 18.0 | 17.0 | 1.06 | 1 | - | 17.4 |
| *Tragelaphus angasii* | 110.2 | 64.4 | 1.71 | 4 | - | 18.5 |
| *Tragelaphus buxtoni* | 232.0 | 166.7 | 1.39 | 8 | 11.8 | - |
| *Tragelaphus derbianus* | 680.0 | 440.0 | 1.55 | 20 | - | 20.3 |
| *Tragelaphus eurycerus* | 300.0 | 240.0 | 1.25 | 6 | 10.2 | 21.9 |
| *Tragelaphus imberbis* | 95.6 | 62.1 | 1.54 | 5 | - | 19.8 |
| *Tragelaphus oryx* | 647.3 | 415.8 | 1.56 | 45 | - | 26.1 |
| *Tragelaphus scriptus* | 49.7 | 31.1 | 1.60 | 2 | 7.0 | 15.3 |
| *Tragelaphus spekii* | 102.3 | 60.2 | 1.70 | 2 | 22.6 | 22.1 |
| *Tragelaphus strepsiceros* | 240.8 | 159.2 | 1.51 | 16 | 20.8 | 23.5 |
